# Severe Early Congenital Syphilis with Multiorgan Involvement in a Preterm Neonate: A Case Report

**DOI:** 10.3390/reports9030214

**Published:** 2026-07-08

**Authors:** Iva Prodanova, Preslava Gatseva, Hristiana Delvarska, Todor Vasilev, Victor Donev

**Affiliations:** 1 Department of Obstetrics and Gynecology, Medical University Pleven, 5800 Pleven, Bulgaria; 2 Clinic of Neonatology, Dr. Georgi Stranski University Hospital, 5800 Pleven, Bulgaria; 3 Heart and Brain Hospital, 5800 Pleven, Bulgaria

**Keywords:** congenital syphilis, neonate, prematurity, multiorgan involvement, pneumonia, vertical transmission

## Abstract

**Background and Clinical Significance:** Lues remains a global health concern despite the well-known nature of its symptoms, the availability of diagnostic methods, and the existence of effective therapy. The recent increase in maternal syphilis has been accompanied by a rise in congenital infections, which are associated with stillbirth, prematurity, neonatal mortality, and severe multisystemic disorder. In newborns, it may present with highly variable clinical manifestations, making timely diagnosis and treatment essential. We report a case of severe early congenital syphilis in a premature newborn with extensive multiorgan involvement; **Case Presentation:** We present a case of a male infant born at 31 + 6 weeks of gestation to a 26-year-old mother with inadequate antenatal care and no documented screening or treatment for syphilis during pregnancy. Prenatal ultrasound revealed fetal ascites. At birth, the infant presented with severe respiratory failure requiring immediate resuscitation, endotracheal intubation, and intensive care support. Clinical findings included hepatosplenomegaly, generalized edema, ascites, petechial rash, palmoplantar desquamation, severe thrombocytopenia, anemia, coagulopathy, liver dysfunction, and hemorrhagic syndrome. Maternal and neonatal serologic testing confirmed syphilis infection. The clinical course was complicated by pneumonia with prolonged mechanical ventilation, cardiovascular involvement impairing cardiac function, and heart failure. Treatment consisted of intravenous penicillin G, broad-spectrum antimicrobial therapy, antifungal medication, respiratory support, transfusion therapy, cardiovascular management, and intensive multidisciplinary care; **Conclusions:** This report presents consequences of untreated maternal syphilis and underscores the importance of timely diagnosis, early initiation of penicillin therapy, and close multidisciplinary follow-up to optimize outcomes in neonates.

## 1. Introduction and Clinical Significance

Syphilis is an ancient infectious disease caused by the spirochete *Treponema pallidum*. It is believed that sexually transmitted syphilis emerged around 3000 BC, with the pathogen undergoing multiple genetic adaptation and evolutionary changes over time [1]. Despite advances in public health the disease continues to circulate among sexually active populations worldwide.

Maternal–fetal transmission occurs primarily via the transplacental (vertical) route, when spirochetes travers the placental barrier through the maternal bloodstream. Less commonly, infection may occur during delivery through direct contact with infectious genital lesions [2]. The risk of fetal infection exists at any stage of pregnancy but it peaks during early maternal syphilis (primary and secondary stages), reaching up to 100%, and declines as the disease progresses. If pregnancy occurs more than one year after maternal infection, the risk decreases to approximately 10–30% [3].

Over the past decade, the global burden of syphilis has increased significantly, from approximately 6 million new cases in 2015 to 9 million in 2025. Congenital syphilis is now the second leading cause of stillbirth worldwide [4]. It is also associated with preterm birth, small-for-gestational-age infants, and increased neonatal mortality. Clinical manifestations in newborns are highly heterogenous, ranging from asymptomatic infection to severe multisystem disease [5].

This report describes a premature infant with severe early congenital syphilis and extensive multisystem involvement, illustrating how missed antenatal screening and treatment may lead to life-threatening neonatal disease.

## 2. Case Presentation

We present the case of a premature newborn with early congenital syphilis and severe multiorgan involvement. A summary of the key clinical events is presented in Table 1. A preterm male infant was born at 31 + 6 weeks of gestation by spontaneous vaginal delivery, from a sixth pregnancy and fourth birth to a 26-year-old mother from a socially disadvantaged ethnic minority. The pregnancy had not been adequately monitored, and there was no documented evidence of appropriate antenatal screening or treatment for maternal syphilis. The mother reported bacteriuria of unclear etiology. There was spontaneous rupture of membranes of unknown duration with the presence of thick yellow-green amniotic fluid. Ultrasound examination at admission revealed fetal ascites and suggested possible congenital anomalies.

The newborn had the following anthropometric parameters: birth weight 2400 g (90th percentile), length 44 cm (90th percentile), and head circumference 31 cm (approximately 90th percentile). According to the Fenton growth chart for boys, 2023, the infant was large for gestational age. Initial neonatal adaptation was severely compromised, with an Apgar score of 2 at 1 min, accompanied by bradycardia and absence of spontaneous respiratory efforts, requiring immediate resuscitation and endotracheal intubation. The patient was subsequently admitted to the Neonatal Intensive Care Unit (NICU) and placed on conventional mechanical ventilation.

**Figure 1:**
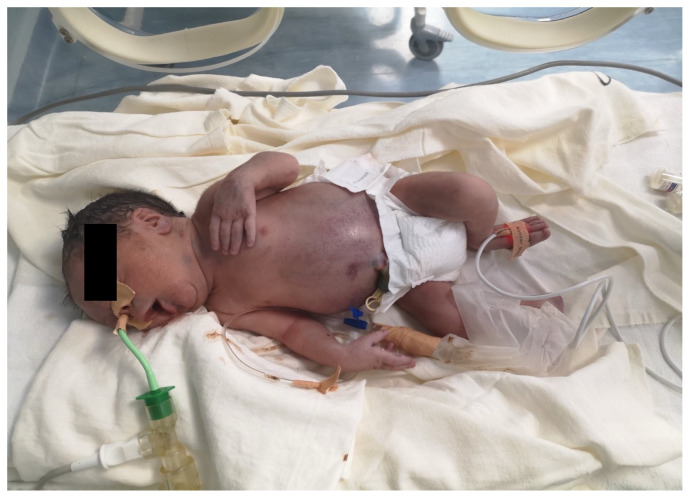
Clinical photograph of the patient at birth showing abdominal distension with livid skin discoloration. Petechiae visible on face, abdomen, and extremities.

**Figure 2:**
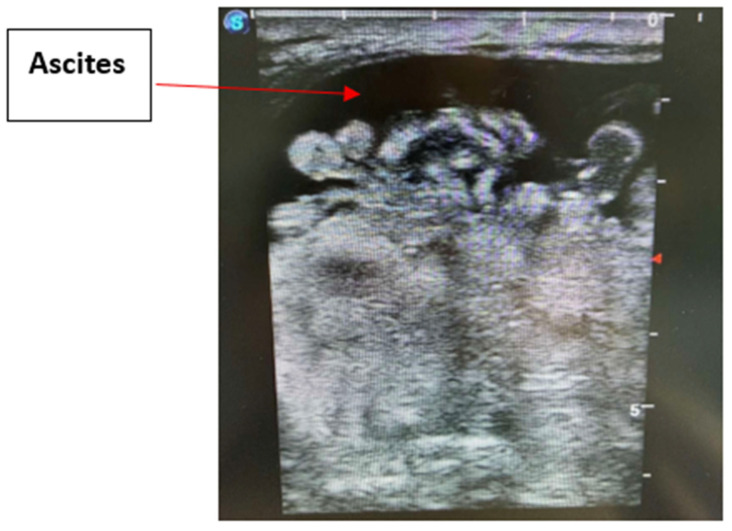
Abdominal ultrasound with free fluid—ascites.

Clinical examination on admission to the ward revealed generalized hypotonia, cyanosis, petechial rash on the face, trunk, and extremities. The palms and soles showed erythema, lamellar desquamation, and isolated erosions (Figure 1). The abdomen was markedly distended with livid skin discoloration and hepatosplenomegaly. Pubic area was also edematous with bilateral hydrocele. Ultrasound scan confirmed ascites (Figure 2).

**Figure 3:**
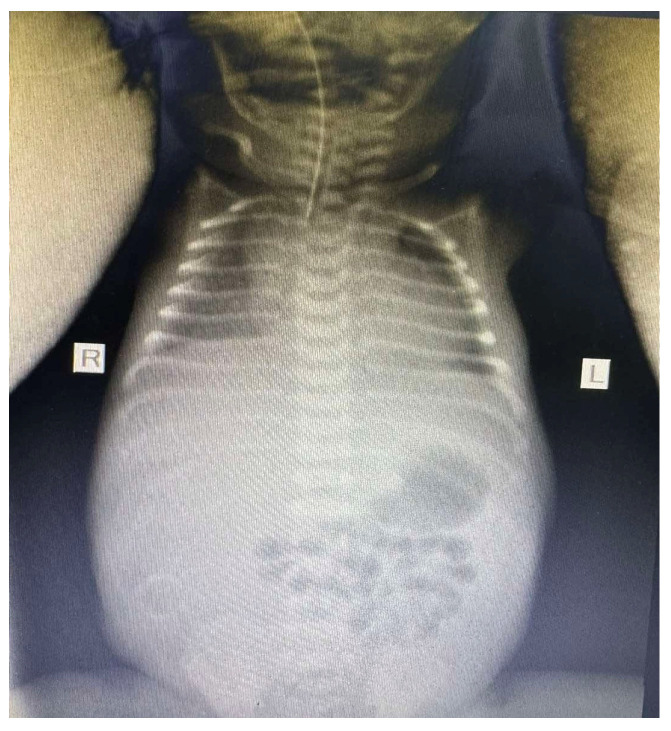
Chest and abdominal X-ray demonstrating neonatal respiratory distress syndrome (NRDS) and hepatosplenomegaly.

Due to severe respiratory distress syndrome requiring maximal ventilatory support, exogenous surfactant was administered, with only transient clinical improvement (Figure 3). The infant was in a state of shock with hypotension, anuria, and fever. A low-dose dopamine infusion was initiated along with normal saline boluses. Transient acute kidney injury resolved within three days.

**Figure 4:**
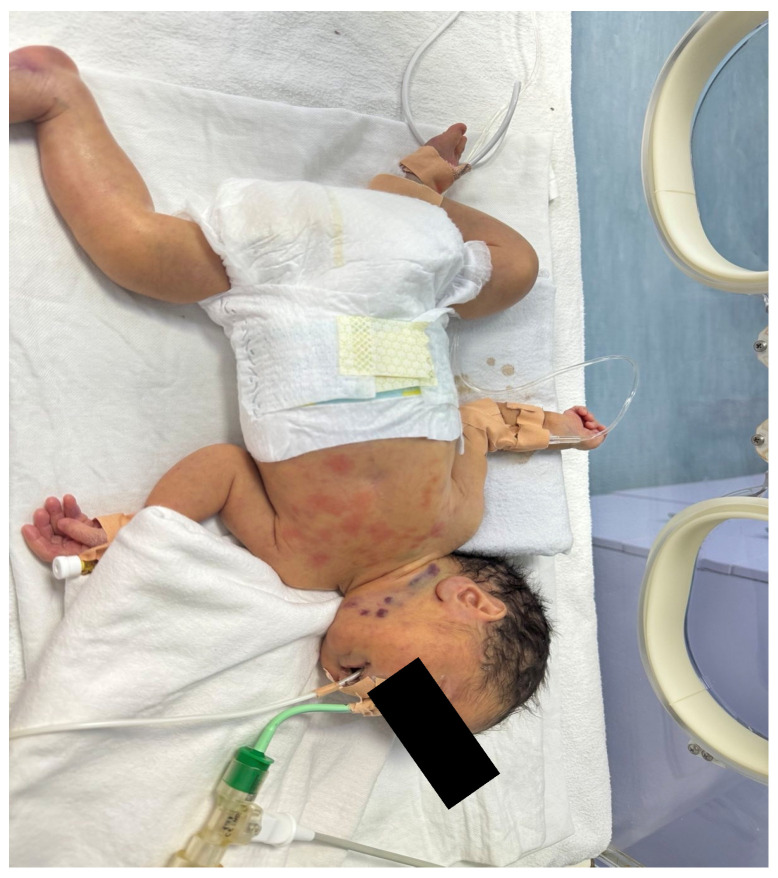
Clinical photograph of the patient (day 3) demonstrating spontaneous subcutaneous hemorrhages: petechiae, suffusions, and ecchymoses.

Initial laboratory evaluation demonstrated severe thrombocytopenia (31 × 10^9^/L), anemia, leukocytosis, and increased C-reactive protein (Table 2). Empirical antibiotic therapy (Ampicillin and Gentamicin) was initiated for presumed sepsis. Within a few hours after birth, a hemorrhagic syndrome developed, manifested by petechiae, suffusions, profuse bleeding from the umbilical stump, and hematemesis (Figure 4). Given the clinical condition and symptomatology during the first 24 h of life, emergency treatment with blood products was administered, including platelets, fresh frozen plasma, and packed red blood cells. Management of the hemorrhagic syndrome was supplemented with vitamin K and etamsylate. Hypoproteinemia required transfusion of human serum albumin. A choleretic agent was introduced to address liver dysfunction. Biochemical analysis and coagulation studies on the second day after birth revealed liver dysfunction characterized by elevated transaminases, hypoalbuminemia, and elevated D-dimer, consistent with hepatic involvement and systemic inflammatory response (Table 3).

On the second postnatal day, maternal serologic testing was positive for syphilis, with a rapid plasma reagin (RPR) titer of 1:32 and strongly reactive treponemal testing (TPHA 3+). Neonatal serology revealed an RPR titer of 1:32 and positive treponemal antibodies. In the context of compatible clinical findings and laboratory abnormalities, these results supported the diagnosis of early congenital syphilis. Additional investigations were performed to evaluate potential complications and differential diagnoses. Transfontanelle ultrasonography showed no evidence of intracranial abnormalities. Newborn hearing screening using otoacoustic emissions was passed bilaterally. Serologic testing for human immunodeficiency virus (HIV) was negative. Histological examination revealed placentomegaly, fibrosis of the chorionic villi, fibrin deposition in the intervillous spaces, and decidua with marked leukocytic infiltration, abscess formation, and fibrinoid necrosis.

Following dermatovenereologic consultation, antimicrobial therapy was changed to intravenous penicillin G (90,000 IU four times daily). Because of the patient’s critical clinical condition, leukocytosis, elevated C-reactive protein levels, and the inability to exclude concomitant bacterial sepsis, treatment with meropenem (50 mg twice daily intravenously) and vancomycin (25 mg twice daily intravenously) was also initiated. After isolation of *Candida albicans* from gastric aspirate antifungal therapy was initiated.

**Figure 5:**
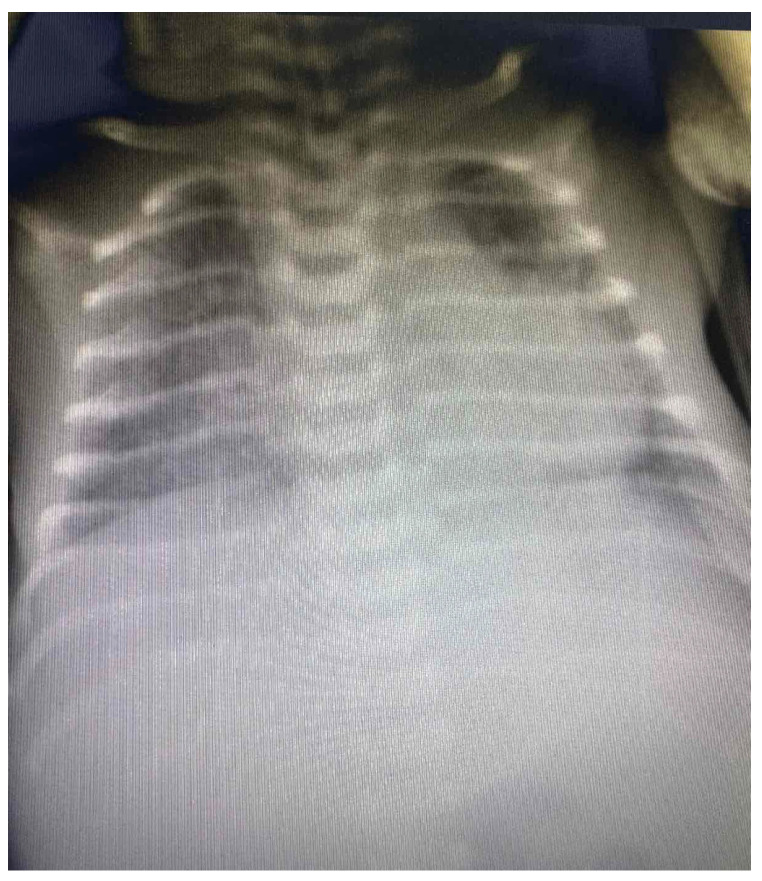
Chest X-ray revealed total opacification of the lungs, radiologically compatible with pneumonia alba.

**Figure 6:**
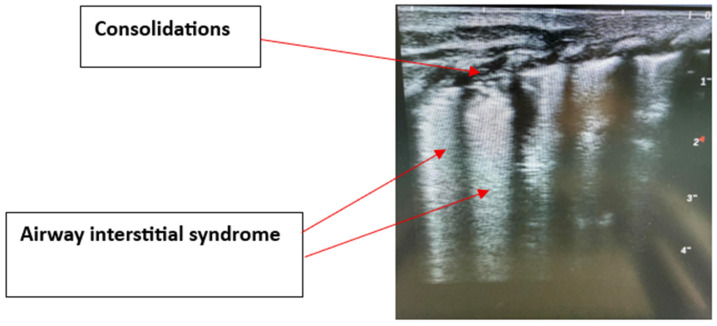
Lung ultrasound demonstrating consolidations and airway interstitial syndrome.

During the second postnatal week, severe respiratory failure persisted, and the infant was still intubated on ventilatory support. Chest x-ray demonstrated reduced pulmonary transparency with a “ground-glass” appearance (Figure 5). Lung ultrasonography revealed consolidations and airway interstitial syndrome (Figure 6). The clinical course and imaging findings were clinically and radiologically compatible with pneumonia alba. Definite extubation was successfully achieved on postnatal day 19, after which the infant remained in an oxygen-enriched environment until respiratory stabilization.

**Figure 7:**
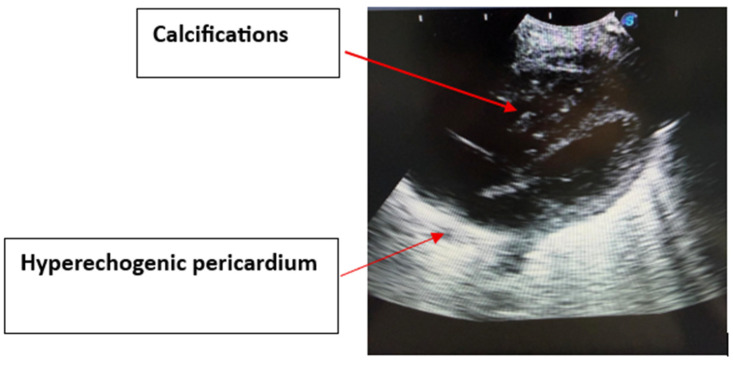
Echocardiography PLAX view.

During the second week, a systolic cardiac murmur of grade 2/6 was detected. Pediatric cardiology consultation and echocardiography revealed persistent fetal communications—patent foramen ovale and patent ductus arteriosus, pericardial separation of up to 5 mm and hyperechogenicity, suggestive of prior in utero fibrinous pericarditis (Figure 7). A recommendation for pharmacological closure of the patent ductus arteriosus was made, and after assessment of laboratory parameters and clinical condition, a three-day course of ibuprofen was administered. The patient was re-evaluated by a pediatric cardiologist before discharge. Persistent interatrial communication was noted, while the ductus arteriosus was no longer visualized. Calcifications along the endocardium of the right ventricle were observed, along with grade 1 tricuspid insufficiency, impaired cardiac pump function, and heart failure. A diuretic and an ACE inhibitor were added to the treatment regimen.

Antibiotic treatment with penicillin G continued for a total of 24 days [6]. Although this exceeded the standard recommended duration, the prolonged treatment course was based on the recommendation of the consulting dermatovenereologist and the decision of the neonatal care team because of the patient’s severe multisystem disease, persistent clinical manifestations, and ongoing inflammatory activity. Meropenem and vancomycin were administered for 14 days. Therapy was discontinued after resolution of clinical and laboratory evidence of systemic inflammation and sterile microbiological cultures from tracheal aspirate, external ear secretion, feces, and blood cultures.

At one month of age, the patient was in satisfactory condition and presented with neonatal conjunctivitis (purulent ocular discharge) treated with topical antibiotic drops, persistent cardiac murmur, and oxygen dependence. Nebulizations with beta-agonist and corticosteroid were prescribed due to developed anemia of prematurity necessitating blood transfusions twice (Table 4).

The infant remained in the NICU for 53 days until complete resolution of respiratory failure. Cranial ultrasound scan at term equivalent age was unremarkable. No ophthalmologic examination, cerebrospinal fluid analysis, or long-bone radiography was performed during hospitalization because of the patient’s critical clinical condition and the need to prioritize intensive care interventions. Laboratory parameters at discharge are presented in Table 5.

Following comprehensive intensive therapy, the patient was discharged in good physical condition with ongoing cardiotonic treatment. A detailed follow-up plan was provided to the caregivers, including serologic reassessment (RPR) at 6 months of age, neurodevelopmental follow-up by a pediatric neurologist, cardiology, pulmonology, ophthalmologic, and orthopedic evaluations.

## 3. Discussion

Congenital syphilis remains a significant global public health problem despite the availability of effective screening and treatment strategies. The increasing incidence of maternal syphilis reported worldwide over the last decade has been accompanied by a parallel rise in congenital infections. According to the World Health Organization (WHO), in 2022, there were an estimated 700,000 cases of congenital syphilis and 390,000 adverse birth outcomes globally, 53% of which occurred in women who attended antenatal care but were not screened for syphilis [7]. The presented case illustrates the severe clinical course that may occur when maternal infection remains undiagnosed and untreated during pregnancy. Vertical transmission of Treponema pallidum can occur at any stage of gestation, with the highest risk during primary and secondary stages of maternal infection due to high spirochetemia [8].

Although several national regulations in Bulgaria mandate screening of all pregnant women for syphilis, the European Centre for Disease Prevention and Control reports that during the period 2014–2023, our country recorded the highest number of congenital syphilis cases in the European Union [9,10,11,12]. The absence of documented prenatal screening and treatment in this case likely contributed to transplacental transmission and the development of severe early congenital disease. Placental pathology, demonstrating an enlarged placenta, villous fibrosis, and inflammatory infiltrates, further supports intrauterine infection and chronic placental inflammation, findings that have been frequently described in congenital syphilis [13]. Furthermore, the presence of fetal ascites detected prenatally suggests that the infection may have been established in utero for a prolonged period, leading to progressive multisystem involvement antenatally.

The diagnosis in the present report was supported by compatible clinical manifestations, positive maternal and neonatal serologic findings, placental histopathological abnormalities consistent with congenital infection, and a favorable clinical response to penicillin therapy. Although direct microbiological confirmation of Treponema pallidum was not available, according to current CDC recommendations, this patient was classified as a highly probable case of congenital syphilis. Direct detection of Treponema pallidum is not required for the diagnosis or initiation of treatment [6].

Clinical presentation is highly variable, ranging from asymptomatic infection to severe systemic illness. The neonate in this report presented with a constellation of findings, characteristic of severe early congenital syphilis, including hepatosplenomegaly, petechial rash, palmoplantar desquamation, hematologic abnormalities, and respiratory compromise. Hematologic manifestations such as thrombocytopenia and anemia are among the most common laboratory abnormalities in affected neonates and are often associated with hemorrhagic complications, as observed in our patient. In addition, hepatic involvement manifested by jaundice, elevated transaminases, and coagulopathy reflects the systemic dissemination of *T. pallidum* and the resulting inflammatory response [14].

The differential diagnosis included neonatal sepsis and other congenital infections, particularly those included in the TORCH spectrum. Clinical features such as hepatosplenomegaly, thrombocytopenia, jaundice, rash, and respiratory distress may be observed in several congenital infectious diseases and are not specific to congenital syphilis. However, the combination of characteristic dermatologic findings, positive maternal and neonatal serologic tests, placental histopathological abnormalities consistent with congenital infection, and the favorable response to penicillin therapy strongly supported the diagnosis of early congenital syphilis. In addition, HIV serology was negative, and no clinical or laboratory findings suggestive of an alternative congenital infection were identified during hospitalization.

One of the most severe complications was the development of profound respiratory failure with clinical and radiological findings compatible with pneumonia alba. However, the coexistence of prematurity and neonatal respiratory distress limits the ability to definitively distinguish this condition from respiratory distress syndrome, bacterial pneumonia, or pulmonary edema [15]. The prolonged need for mechanical ventilation in our patient underlines the potential severity of pulmonary involvement in congenital syphilis.

Cardiovascular manifestations are less commonly emphasized in congenital syphilis and more often include invasion of the vasa vasorum, inflammation of the arterial wall, and formation of aneurysms, aortic regurgitation, and ventricular hypertrophy. The echocardiographic findings in our case, including endocardial calcifications and impaired cardiac function, may reflect prior intrauterine inflammatory injury. This is a rarely reported finding in the literature [16,17].

Similar cases of severe early congenital syphilis with multisystem involvement have been reported in the literature [18]. However, the combination of profound hemorrhagic manifestations, prolonged respiratory failure clinically compatible with pneumonia alba, and unusual cardiac findings observed in our patient appears to be uncommon. This case further illustrates the broad clinical spectrum and potential severity of congenital syphilis in premature infants.

Prematurity likely contributed significantly to the severity of the clinical course. Preterm infants more frequently develop infections, sepsis, anemia, and coagulation disorders due to the immaturity of the immune system and all other organs and systems [19].

The management of congenital syphilis is challenging for neonatologists. In our patient, early initiation of high-dose intravenous penicillin G was used to control the infection. Given the severe clinical presentation and the inability to exclude concurrent bacterial sepsis, broad-spectrum antimicrobial therapy was also administered initially. The prolonged intensive care course required a multimodal approach, including respiratory support, transfusion therapy, management of coagulopathy, treatment of cholestasis, and cardiovascular stabilization. This case highlights the complexity of care often required in neonates with severe congenital infections.

Several limitations should be acknowledged. Direct detection of Treponema pallidum by molecular or histopathological methods was not available. Lumbar puncture, long-bone radiography, and formal ophthalmologic examination were not performed during hospitalization; therefore, central nervous system, skeletal, and ocular involvement could not be definitively excluded. In addition, the exact timing and stage of maternal infection could not be determined because of the lack of adequate prenatal surveillance. Serologic testing was performed 14 days after treatment initiation and demonstrated persistent reactivity, which was expected given the natural decline of passively transferred maternal antibodies and the prolonged serologic response observed in congenital syphilis. Follow-up serologic assessment (RPR) at 6 months of age, as well as neurological, cardiopulmonary, ophthalmologic, and orthopedic evaluations, were recommended; however, the corresponding data were not available to the authors because these assessments were performed in other healthcare institutions.

## 4. Conclusions

In conclusion, this case demonstrates that congenital syphilis can present as a severe multisystem disease in the neonatal period, particularly in premature infants born to mothers without adequate antenatal care. It is not merely a result of missed therapy, but rather a consequence of the lack of effective integration between social and health structures and practices. Early recognition of clinical signs, prompt serologic testing, immediate initiation of appropriate therapy, and a multidisciplinary approach are essential for improving outcomes. The responsibility for controlling and reducing the incidence of the disease is shared among institutions, specialists, and society.

## Figures and Tables

**Table 1:** Clinical Timeline of Key Events.

	Clinical Timeline of Key Events.
First 24 h	Admission to NICU; Initiation of mechanic ventilation; Immediate resuscitation
Day 2	Maternal and neonatal syphilis confirmed
Day 2	Penicillin G started
Day 1, 2 and 16	Surfactant administration
First 4 days and day 21	Transfusions: RBC, FFP, Platelets and Human serum albumin 20%
Day 19	Extubation
Day 53	Discharge

**Table 2:** Complete blood count (CBC) and C-reactive protein (CRP) levels during the first three postnatal days.

Test	Values at Birth	Values at Day 2	Values at Day 3
Hb (g/L)	89	58	138
Hct	0.24	0.25	0.39
Er (×10^12^/L)	2.36	2.4	3.93
Thr (×10^9^/L)	31	58	51
Leu (×10^9^/L)	33.5	30.7	34.8
CRP	37	20.9	-

**Table 3:** Biochemical and Coagulation Parameters on the Second Neonatal Day.

Day	LDH	ASAT	ALAT	GGT	AP	ALB	Na	Cl	K	Ca	iCa	PT [%]	aPTT [s]	Fb [g/L]	D-Dimer [mg/L]
2	97	23	68	188.1	287	22.6	138	101	5.1	1.99	1.19	97	23	2.6	>10

Abbreviations: Hb, hemoglobin; Hct, hematocrit; Er, erythrocytes (red blood cell count); Thr, thrombocytes (platelet count); Leu, leukocytes (white blood cell count); CRP, C-reactive protein; LDH, lactate dehydrogenase; ASAT (AST), aspartate aminotransferase; ALAT (ALT), alanine aminotransferase; GGT, gamma-glutamyl transferase; AP (ALP), alkaline phosphatase; ALB, albumin; PT, prothrombin time; aPPT, activated partial thromboplastin time; Fb, fibrinogen; D-dimer [mg/L].

**Table 4:** Complete blood count (CBC) values at one month and five days of age and following blood transfusion.

Test	Values at One Month and 5 Days	Values After Haemotransfusion
Hb (g/L)	99	147
Hct	0.28	0.43
Er (×10^12^/L)	3.15	4.78
Thr (×10^9^/L)	218	194
Leu (×10^9^/L)	7	9.4

**Table 5:** Hematological and Biochemical Parameters at Discharge.

Test	Values at Discharge	Liver Enzymes	Values at Discharge
Hg (g/L)	135	LDH	408
Hct	0.38	ASATA	36
Er (×10^12^/L)	4.3	ALAT	71
Thr (×10^9^/L)	332	GGT	104.6
Leu (×10^9^/L)	9.6	AP	467
		TP (g/L)	53.8

Abbreviations: Hb, hemoglobin; Hct, hematocrit; Er, erythrocytes (red blood cell count); Thr, thrombocytes (platelet count); Leu, leukocytes (white blood cell count); LDH, lactate dehydrogenase; ASAT (AST), aspartate aminotransferase; ALAT (ALT), alanine aminotransferase; GGT, gamma-glutamyl transferase; AP (ALP), alkaline phosphatase; TP, total protein.

## Data Availability

The data presented in this study are available on request from the corresponding author. The data are not publicly available due to privacy and to protect patient confidentiality.
